# Immunoregulation of synovial macrophages for the treatment of osteoarthritis

**DOI:** 10.1515/biol-2022-0567

**Published:** 2023-01-31

**Authors:** Mingze Xu, Yunhan Ji

**Affiliations:** Department of Orthopedics, Shanghai Tongren Hospital, Shanghai Jiao Tong University School of Medicine, Shanghai, 200336, P. R. China

**Keywords:** osteoarthritis, synovitis, macrophage, inflammation, polarization, immunoregulation

## Abstract

Osteoarthritis (OA) is the most common joint disease affecting approximately 10% of men and 18% of women older than 60. Its pathogenesis is still not fully understood; however, emerging evidence has suggested that chronic low-grade inflammation is associated with OA progression. The pathological features of OA are articular cartilage degeneration in the focal area, including new bone formation at the edge of the joint, subchondral bone changes, and synovitis. Conventional drug therapy aims to prevent further cartilage loss and joint dysfunction. However, the ideal treatment for the pathogenesis of OA remains to be defined. Macrophages are the most common immune cells in inflamed synovial tissues. In OA, synovial macrophages undergo proliferation and activation, thereby releasing pro-inflammatory cytokines, including interleukin-1 and tumor necrosis factor-α, among others. The review article discusses (1) the role of synovial macrophages in the pathogenesis of OA; (2) the progress of immunoregulation of synovial macrophages in the treatment of OA; (3) novel therapeutic targets for preventing the progress of OA or promoting cartilage repair and regeneration.

## 1 Introduction

Osteoarthritis (OA) is the most prevalent form of arthritis and a major cause of disability worldwide, affecting an estimated 10% of men and 18% of women over 60 [1,2]. However, due to the unclear pathogenesis, few treatments are currently available to prevent the onset or progression of OA. Compared to earlier paradigms, OA is now recognized as a low-grade inflammatory disease affecting the entire joint. It is characterized by articular cartilage destruction, subchondral bone remodeling, osteophyte formation, and synovium inflammation (synovitis) [3].

A recent single-cell RNA-seq study has identified various synovial joint immune cell types and characterized their dynamic alterations during the pathological progression of post-traumatic OA in mouse knee joints following anterior cruciate ligament (ACL) rupture [4]. Multiple immune cell types in joints were detected, including neutrophils, monocytes, macrophages, B cells, T cells, natural killer (NK) cells, and dendritic cells. The monocyte and macrophage populations showed the most dramatic changes after injury. Further characterization of monocytes and macrophages revealed nine major subtypes with distinct transcriptomic signatures, including two macrophage populations with phagocytic genes and enrichment of growth factors [4].

Studies have also found that during the development of OA, many matrix-degrading enzymes, such as matrix metalloproteinases (MMPs), are significantly upregulated. The increased secretion of pro-inflammatory cytokines indicated that the synovium undergoes an inflammatory process, leading to the degradation of the cartilage matrix [5]. Furthermore, increasing evidence suggests that persistent low-grade synovial inflammation exacerbates cartilage damage [6], where synovial macrophages have a critical role [7]. Therefore, immunoregulation of macrophages might limit the pro-inflammatory effects and promote anti-inflammatory effects of synovial macrophages, restoring the normal composition of the extracellular chondrocyte matrix and promoting cartilage repair, which in turn improves joint function and facilitate daily activities of patients with OA [8,9,10,11].

The present review discussed the following: (1) the role of synovial macrophages in the pathogenesis of OA; (2) the progress of immunoregulation of synovial macrophages in the treatment of OA; (3) novel therapeutic targets for preventing the progress of OA or promoting cartilage repair and regeneration.

## 2 Role of synovial macrophages in OA

**Figure 1:**
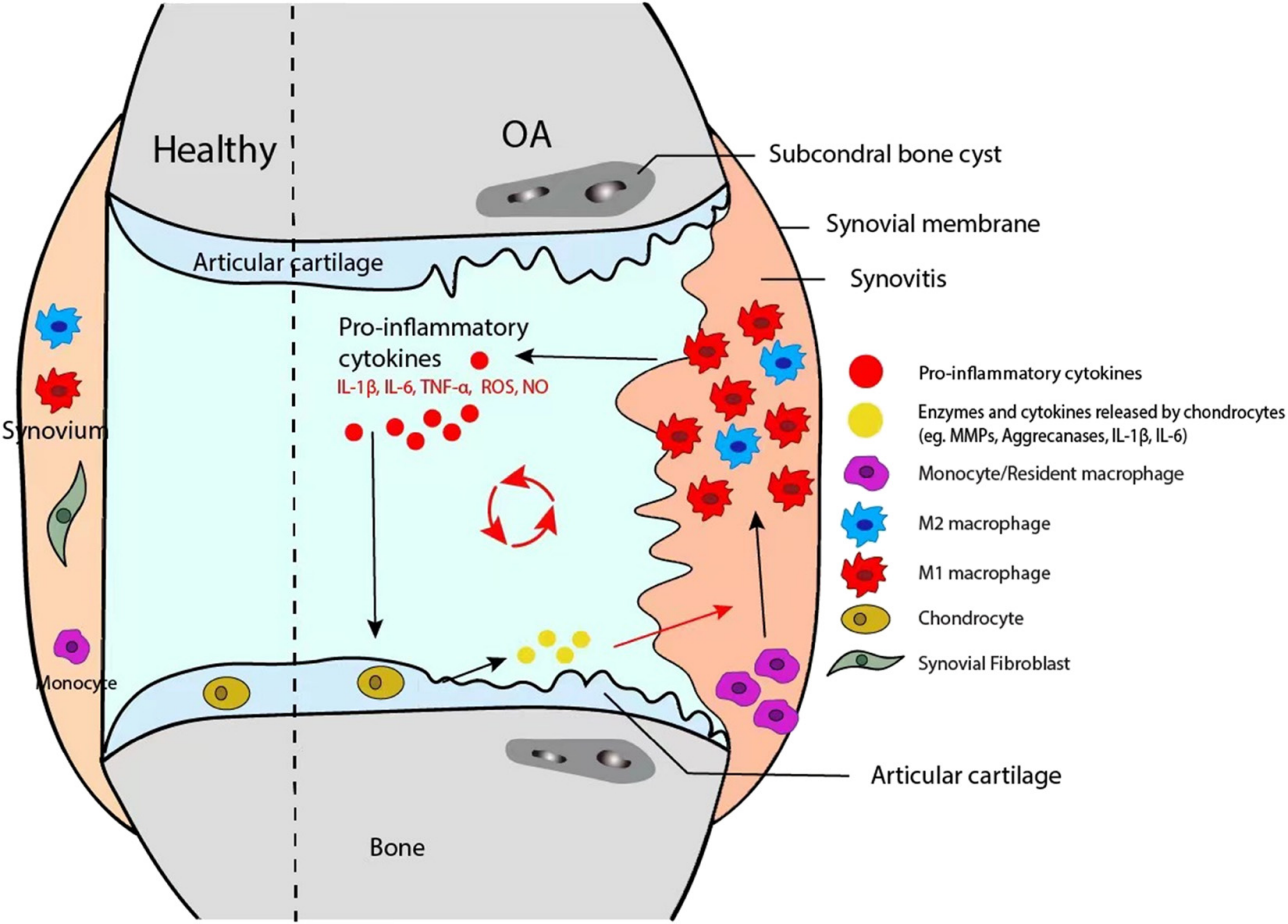
Mechanisms of macrophages in the pathogenesis of OA. Microenvironment stimuli promote synovial macrophage activation and polarization. M1-polarized macrophages in the synovium contribute to OA by releasing pro-inflammatory cytokines that lead to inflammation and subsequent cartilage degradation and osteophyte formation. Polarized macrophages alter the intercellular signaling pathways in chondrocytes, promoting the degradation of extracellular matrix (ECM) components. ECM, in turn, acts as DAMPs and further stimulates macrophage activation and polarization, resulting in a repeating cycle of inflammation and cartilage degradation. Polarized synovial macrophages and macrophage reprogramming could provide therapeutic targets for OA patients.

Different studies have reported on the role of macrophages in the pathogenesis of OA. In normal synovium, macrophages are the predominant cell type [12]. Synovial macrophages are found on the surface of the synovial membrane in healthy joints, providing regulatory factors for cartilage and bone turnover. Similar to other tissue-resident macrophages, they may also remove cell debris and pathogens to prevent sterile and septic inflammation [13]. Increasing evidence highlights the impact of synovitis and macrophage activation on the occurrence and development of OA [14]. A previous study suggested that monocyte/macrophages are the most abundant immune cells in the synovial fluid of OA patients, accounting for ∼36.5% of the total leukocyte. They are also the CD14 + CD16 + double-positive pro-inflammatory cells [15]. Histological studies have also observed more diffusely distributed macrophages in the synovial lining of OA [12]. Yet, studies have also found that alteration in their functionalities may alter the joints of OA patients. It was reported that macrophages produce various cytokines, including interleukin-1 (IL-1) and tumor necrosis factor-alpha (TNF-α) in the OA synovium [16]. In addition, cytokines such as granulocyte-macrophage colony-stimulating factor (GM-CSF) and interferon-γ (IFN-γ) produced at the site of inflammation can recruit and activate macrophages [17]. This vicious circle of macrophage activation and pro-inflammatory cytokines production causes deterioration of the inflammatory process and cartilage degradation [18] (Figure 1).

The inflammation-targeted treatment has been confirmed to be effective in alleviating the symptoms of OA [19,20]. Inflammation is a predominant risk factor for OA, which can also affect the function of macrophages. The activation and aging of macrophages affect different processes [21], including Toll-like receptor signal transduction [22,23], phenotypic alterations [24,25], phagocytosis [26,27], and wound repair [24].

### 2.1 Activation of macrophages

**Figure 2:**
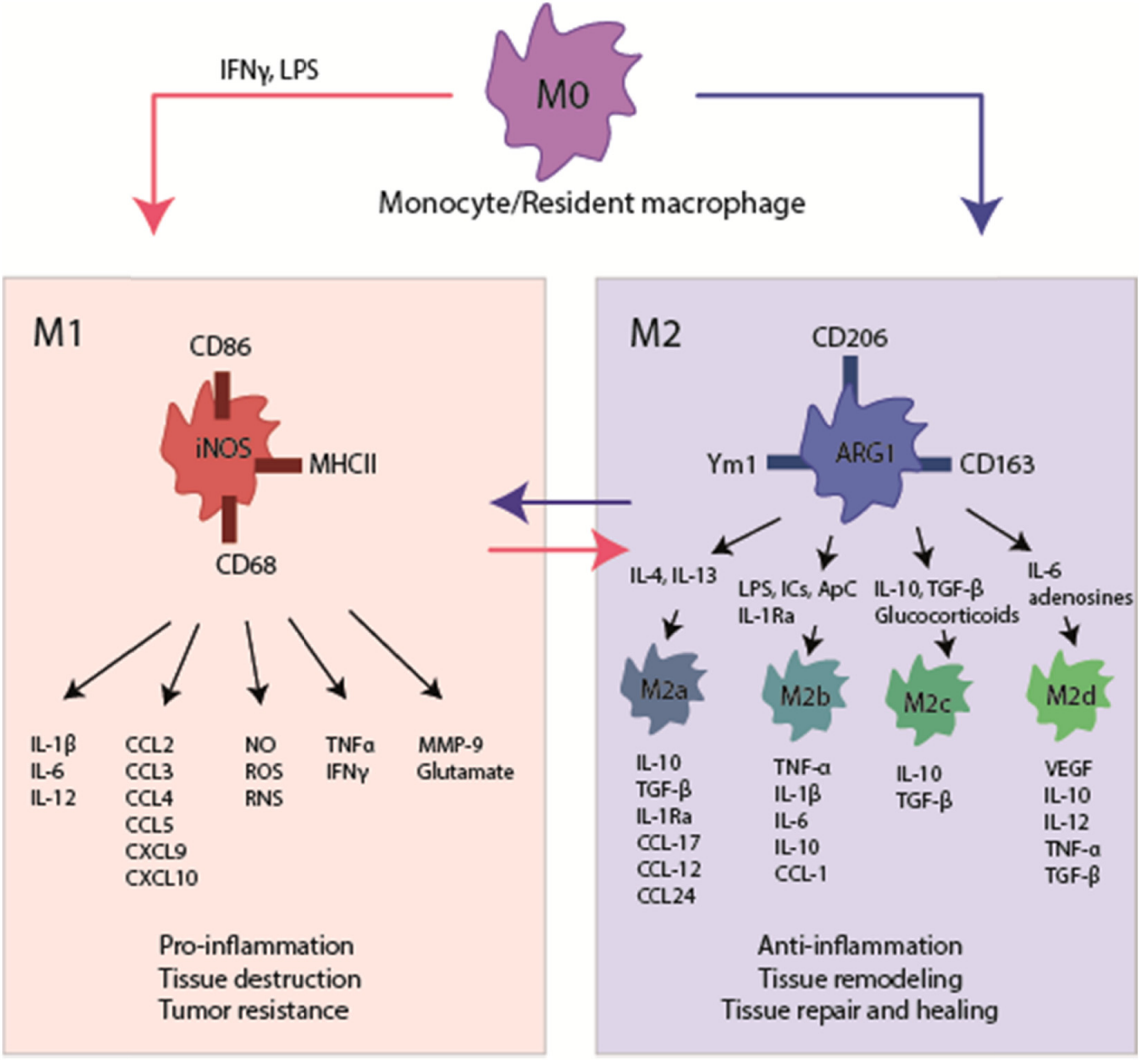
Schematic representation of macrophage activation and polarization. M1 macrophages (or classical activation pathway) are induced by IFN-γ, LPS, or TNF-α; it promotes the immune response by upregulating pro-inflammatory factors TNF-α and IL-1, and downregulating anti-inflammatory factors such as interleukin 10 (IL-10). M2 macrophages (or alternative activation pathway) have four subpopulations: IL-4- and IL-13-induced M2a macrophages, expressing MRC1 and IL-10; M2b macrophages induced by immune complex signaling, expressing IL-10 and major histocompatibility complex class II; M2c macrophages induced by IL-10 and glucocorticoids, expressing MRC1, IL-10 and TGF-β; M2d macrophages can overexpress vascular endothelial growth factor and inducible nitric oxides synthase (iNOS), or lower expression of TNF-α and arginase 1 (arginase 1, Arg1) and participate in angiogenesis and wound healing. Among them, M2a macrophages are mainly related to anti-inflammatory activity, and M2c macrophages are mainly related to tissue repair.

Under healthy conditions, macrophages dynamically and regularly adjust their phenotype and function to stabilize the immune system. However, during pathological conditions, a certain phenotype of macrophages predominates and persists, which is a phenomenon also known as polarization of macrophages [28] (Figure 2).

M1 macrophages can be induced by IFN-γ, lipopolysaccharide (LPS), or Toll-like receptors (TLRs) through the production of reactive oxygen intermediates such as NO. M1 macrophages kill and clear pathogens through lysosomal enzymes and other pathways. They also secrete a variety of chemokines and pro-inflammatory cytokines, such as IL-1β, IL-6, and TNF-α, to participate in the inflammatory response, tissue damage, and cell destruction [29,30,31] (Figure 2). At this time, the immune balance is destroyed, and the corresponding tissues are damaged due to the acute and, later on, chronic inflammatory reaction. In their study, Liu et al. examined the phenotypic status of macrophages in the peripheral blood and synovial fluid of 80 patients with knee OA and observed that the M1/M2 was significantly higher in these patients than in healthy controls. Also, this change was significantly associated with the OA classification, indicating the special significance of controlling the activation and polarization of macrophages for guiding the treatment of OA [32].

M2 macrophages are induced by IL-4 and IL-13. M2 can release anti-inflammatory cytokines such as transforming growth factor-β (TGF-β) and IL-10, inhibit inflammation, and promote tissue repair (Figure 2). M2 macrophages can be divided into four subpopulations [33]: IL-4 and IL-13-induced M2a macrophages, expressing mannose receptor C-type 1 (MRC1) and IL-10; M2b macrophages induced by immune complex signaling, expressing IL-10 and major histocompatibility complex class II; M2c macrophages induced by IL-10 and glucocorticoids, expressing MRC1, IL-10 and TGF-β; M2d macrophages can overexpress vascular endothelial growth factor and inducible nitric oxides synthase (iNOS), or lower expression of TNF-α and arginase 1 (arginase 1, Arg1), which have a role in angiogenesis and wound healing [34]. Among them, M2a macrophages are mainly related to anti-inflammatory activity, while M2c macrophages have an important role in tissue repair [33,35]. In addition, in mouse models of arthritis, IL-10 was identified to inhibit the occurrence and progression of arthritis [36,37].

A research group found significantly increased M1-type macrophages in OA patients and mouse models [38]. They used two OA mouse models (M1 or M2 macrophage conditional knockouts) to identify the role of M1- or M2-type macrophages in the development of OA. The mouse model with accumulated synovial M1-type macrophages presented with increased OA score, thinner articular cartilage, increased surface fibrosis areas, abnormal distribution of chondrocytes, and significantly increased volume and surface area of periarticular osteophytes, which exacerbated the progression of OA [38]. On the contrary, the mouse model with accumulated synovial M2-type macrophages presented with decreased synovial inflammation of the injured ACL and decreased OA score and osteophytes, indicating that synovial M2-type macrophage polarization prevents the development of collagenase-induced OA [38]. Moreover, gene sequencing analysis showed that M1-type macrophages promote the progression of OA by secreting pro-inflammatory factors IL-1, IL-6, and TNF-α, and promote hypertrophic chondrocytes differentiation and maturation, leading to degeneration [38]. Another study reported positive macrophage-specific protein MRP14, indicating the activation of macrophages in an OA animal model [39]. Consecutively, the synovial macrophages were depleted to observe the OA progression. As a result, significantly reduced osteophytes improved the stability of the joints and reduced infiltration of fibroblasts and inflammatory cells (by about 50%) [39]. These findings confirmed the participation of synovial macrophages in the pathological process of OA by promoting synovial fibrosis and osteophyte formation.

In conclusion, macrophages have an important role in the inflammatory response of OA. In the early stage of inflammation, M1-type macrophages phagocytose pathogens, while in the later stage of inflammation, M2-type macrophages regulate the inflammatory tissue microenvironment by secreting anti-inflammatory cytokines such as IL-10, which is conducive to the regeneration and repair of cartilage tissue. Therefore, timely changes in the polarization state of macrophages are critical for the resolution of inflammation. Therefore, it is of great clinical significance to deeply explore the molecular mechanism of macrophage polarization and achieve targeted induction of anti-inflammatory M2 macrophage polarization.

### 2.2 Macrophages and synovitis

Although synovial macrophages are the major immune cells in synovial tissues, their role in the pathogenesis of OA remains poorly understood. A few studies have shown synovial macrophages’ abnormal accumulation and phenotypic changes in the OA synovium [40,41]. Compared with healthy synovium, the number of F4/8+ (macrophage marker) cells showed a remarkable increase, with an increased number of iNOS + cells (M1 macrophage marker) and a reduced number of CD206 + cells (M2 macrophage marker) in OA synovium [38]. Up to 90% of patients with end-stage OA have synovitis with the infiltration of CD68-positive macrophages [32]. Other studies suggested greater conspicuous macrophage infiltration in patients with early-stage OA [42]. Also, numerous inflammatory factors and chemokines were found to be elevated in the isolated synoviocytes from minced synovial tissue samples extracted from OA patients [43]. After the depletion of macrophages using anti-CD14-conjugated magnetic beads, TNF-α, IL-1, and other cytokines, including IL-6, IL-8, monocyte chemoattractant protein-1, and MMPs also showed a marked reduction, suggesting that macrophage could secrete pro-inflammatory factors and promote the production of MMPs [16]. Bondeson et al. found that the level of macrophage-secreted pro-inflammatory factor macrophage migration inhibitory factor was positively correlated with the severity of OA-caused pain [16]. Another research group established an *in vitro* model to study the role of synovial macrophages in OA and found that maintaining the stable phenotype of macrophages is essential for preserving the viability of chondrocytes and maintaining the expression levels of cartilage proteoglycan and collagen [44]. They extracted synovial explants from OA patients for *in vitro* culture, treated them with different cytokines to stimulate the phenotypic changes of macrophages, and administered dexamethasone, rapamycin, bone morphogenetic protein-7 (BMP-7) or pravastatin to evaluate the inflammatory state of synovitis. Dexamethasone showed an anti-inflammatory effect by inhibiting M1 macrophages, while rapamycin inhibited the M2 phenotype to enhance the inflammatory response [44]. These data suggest the use of macrophage phenotypic modulation to guide the treatment of joint inflammation, which could, in turn, help to develop novel therapies for delaying the progression of OA.

### 2.3 Macrophages and subchondral bone destruction/repair

The subchondral bone in OA undergoes an uncoupled remodeling process characterized by macrophage infiltration, osteoclast formation, and increased osteoblast activity resulting in local remineralization and bone sclerosis of end-stage OA [45]. Utomo et al. injected clodronate-liposomes to deplete macrophages in the synovium and injected different doses of TGF-β into the knee joint seven days later [46]. In mice without macrophage depletion, osteophytes formed on the inner and outer sides of the patella and femur, while in mice with synovial macrophage depletion, the formed osteophytes were reduced by ∼70% [46]. They also discovered that synovial macrophages could lead to bone morphogenetic protein 2 (BMP-2) and BMP-4 after TGFβ stimulation [46]. These findings suggest that macrophages are a key intermediate factor in TGFβ-induced osteophytes.

Subchondral bone cysts are a common feature in OA [45]. Cysts-derived macrophages promote osteoclast differentiation and contribute to the expansion of OA cysts and osteolysis [47]. Another study reported that the synovial macrophages differentiate into functional osteoclasts, thereby promoting bone resorption and subchondral bone reconstruction [48]. Besides, TNF-α can indirectly induce osteoclast formation by stimulating macrophage differentiation [49]. Furthermore, the M2 polarization of macrophages has been confirmed to be crucial in the regeneration of subchondral bone. In an animal model of the bilateral trochlear cartilage defect, mice were subcutaneously injected with a mixture of chitosan–glycerophosphate and whole blood or serum [50]. This treatment could induce the chemotactic effect of neutrophils and M2 macrophages to concentrate at the injection site and promote trabecular bone repair and bone regeneration by expressing arginase-1 and releasing angiogenic factors [50].

These data suggest macrophages have an important role in the destruction of subchondral bone in OA patients. Therefore, immunoregulation of macrophages, especially polarizing macrophages toward M2 phenotype, might further elucidate the restoring process of subchondral bone.

### 2.4 Macrophages promote articular cartilage degeneration

The activation of MMPs has been identified as one of the important signs of irreversible damage to articular cartilage. Studies have found that synovial macrophages can mediate the expression of MMPs to induce articular cartilage damage [51]. M1 macrophages induce inflammation and degeneration of OA cartilage explants by up-regulating IL-1, IL-6, and MMP-13, while M2 macrophages have no effect [30]. Utomo et al. established an *in vitro* three-dimensional co-culture system to evaluate the interaction between activated macrophages and chondrocytes to understand the progression and treatment of OA [30]. It was observed that in the co-culture of activated macrophages and normal chondrocytes, MMPs and pro-inflammatory cytokines were increased while aggrecan and type II collagen were decreased, similar to the microenvironment of early-stage OA in clinical practice; whereas in the co-culture of activated macrophages and OA chondrocytes, the expression levels of MMPs and pro-inflammatory factors were remarkably higher than those in the co-culture system with normal chondrocytes [30]. These results suggest that the activation of pro-inflammatory macrophages is involved in promoting OA development. They also showed that diseased chondrocytes could aggravate the activation of macrophages.

## 3 Immunomodulatory macrophages in the treatment of OA

### 3.1 Depletion of macrophages

As macrophages are important in the immune pathogenesis of OA, several studies have tried to deplete macrophages to examine their effect on cartilage health and joint integrity. Previous studies found that depleting the synovial macrophages by intra-articular injection of clodronate-loaded liposomes can significantly decrease the expression of MMP-3 and MMP-9 in the synovium and reduce TGF-β-mediated osteophyte formation in the collagenase-induced OA mouse model [51,52]. In another study, anti-CD14 binding magnetic beads were used to achieve the specific depletion of synovial macrophages in OA synoviocytes *in vitro*, resulting in downregulation of the expression MMPs and fibroblasts-produced cytokines, including IL-6, IL-8, and MCP-1 [16]. However, other studies have reported increased synovial inflammation after the depletion of macrophages, which could not prevent the progression of OA [53,54]. Chamberlain et al. showed that compared with the medial collateral ligament (MCL) of untreated rats, the mechanical strength of MCL was decreased in rats with macrophage depletion [55]. These findings demonstrated that depleting macrophages may affect the inflammatory response around the injured joints while inhibiting the function of macrophages could profoundly impact joint inflammation and bone homeostasis after joint injury.

Currently, a variety of *in vitro* or animal models are available to study macrophage depletion. Yet, these technologies cannot precisely target the specific phenotype of macrophages without affecting other bone marrow lineages, such as dendritic cells and neutrophils. Simply depleting macrophages without considering the polarization of macrophages may not permanently address the OA progression.

### 3.2 Immunomodulatory macrophages

The continued existence of the pro-inflammatory M1 macrophages is generally thought to be detrimental to tissue repair, while the anti-inflammatory M2 macrophages can benefit tissue regeneration. Several cell or animal studies have attempted to improve or treat OA using immunomodulatory macrophages, including regulating and targeting specific signaling pathways [38,56], and other interventions such as extracts of traditional Chinese medicine [57,58], anti-inflammatory drugs [8], and mesenchymal stem cell therapy [59]. Studies highlighting the potential targets of macrophage immunomodulation are listed in Table 1.

**Table 1:** Genes and targets of interest on the immunoregulation of macrophages in OA

References	Relevant gene/treatment	Disease model	Genotype	Upregulated cytokines	Downregulated cytokines	Effect on macrophages	Remark
[77]	NFAT5	DMM-induced OA in mice	NFAT5 haplo-insufficient (NFAT5 +/−) mice	CCL2, IL-1β, MMP-13, ADMATS-5	NA	Macrophage infiltration	NA
[78]	Alpha defensin-1	Meniscal/ligamentous injury, rat	Wistar rats	COL2A1, ACN,MMP3, MMP13 and ADAMTS5	NA	Promoting M1 to M2 macrophage polarization via insulin and Toll-like receptor signaling pathway	Candidate treatment
[79]	Artificial M2 macrophages	Injecting papain, mice	Kunming mice	NA	IL-Iβ, IL-6, IL-17		A promising strategy
[80]	Basic calcium phosphate crystals	Macrophage isolated from Human blood monocyte	NA	CXCL9, CXCL10, HIF1a, GLUT1and hexokinase 2	CCL13, MRC1	Promoting M1 macrophage polarization	Potential therapeutic target
[81]	Lumican	Synovial fluid of OA patients	NA	TLR4	NA	Up-regulating primary macrophage activation and polarization towards the M1-like phenotype	NA
[82]	GM-CSF	The collagenase-induced osteoarthritis (CiOA) in mice	C57BL/6 mice	NA	NA	NA	Potential benefits of anti-GM-CSF (and anti-CCL17) mAb therapy in OA
[83]	The E3 ubiquitin ligase, Itch	Post-traumatic OA joints	C57BL/6J mice; Itch global knockout (Itch −/−) mice, macrophage-specific Itch knockout (MΔItch) mice	NA	NF-kB, JNK, and MARK12	Inhibiting macrophage pro-inflammatory polarization	NA
[84]	PTP-001	DMM-induced OA in rat	Rat	NA	MMP-13, TNFa, IL-1b	Inhibiting macrophage polarization	A promising biologic treatment
[85]	IL-4	DMM-induced OA in mice	C57BL/6J, BALB/cJ mice	CD206, CCL24, CCL18	TNFa	Promoting macrophages polarize towards an M2 phenotype	Could provide therapeutic benefit

Glucocorticoids can decrease the CD68 + macrophages in the synovial fluid of patients with symptomatic knee OA and increase the expression of CD163 in synovial macrophages [60]. A decreased number of macrophages were reported in advanced knee OA after intra-articular injection of hyaluronic acid (HA) or methylprednisolone [61]. Mechanistically, HA mainly stimulates the repair process, while corticosteroids mainly reduce inflammation. Another study on dexamethasone found its anti-inflammatory effect on the synovial explants of OA patients. Dexamethasone inhibited the pro-inflammatory M1 macrophages and promoted the anti-inflammatory M2 macrophages in the culture of polarized primary human monocytes [46]. This study also carried out similar experiments using rapamycin, BMP-7, and pravastatin, finding that rapamycin and BMP-7 could enhance the inflammatory response of synovial explants and inhibit M2 macrophages. Moreover, pravastatin did not affect the inflammatory state of synovial explants, though it could inhibit M2 macrophages [46]. In the papain-induced OA rat model, triamcinolone acetonide (TA) intra-articular injection limited the osteophyte formation but could not affect cartilage degeneration or subchondral sclerosis [62]. The results indicated that TA could induce the differentiation of monocytes into M2 macrophages.

In animal models of OA, different traditional Chinese medicine extracts, such as ginsenoside [63] and squid type II collagen [58], have been verified to alter the polarization state of synovial macrophages and alleviate cartilage degradation in OA.

Other treatments, such as TissueGene-C (TG-C), a novel cell-mediated gene therapy, can also immunomodulate macrophages through local transduction of TGF-β1. In a rat model of monosodium iodoacetate, IL-10 and other M2 macrophage markers were increased in the knee joints of the TG-C group compared with the control group, indicating that TG-C could induce an anti-inflammatory microenvironment in the knee joint [64]. Furthermore, stem cell therapy could alleviate OA by regulating macrophage activation [65]. The stem cells are effective in cartilage repair, as they can differentiate in chondrocytes and replace degraded or dead chondrocytes [66]. The potential of mesenchymal stem cells to repair OA has been shown to rely on their ability to immunomodulate macrophages [67]. In osteochondral defect models, human embryonic stem cell-derived exosomes increased intra-articular CD163 + macrophages (M2), decreased CD86 + macrophages (M1), and reduced intra-articular pro-inflammatory cytokines [67].

The CCR2 signaling pathway has long been of interest to the rheumatology research community due to its pronounced pro-inflammatory and chemoattractive effects. As a major chemotactic pathway for monocytes, the CCL2/CCR2 axis is critical for recruiting CCR2-expressing circulating monocytes to sites of inflammation. However, studies in Ccr2-null mice reported controversial data in terms of mitigating OA. Miller et al. found severe allodynia and structural knee joint damage in ccr2-null mice equal to wild-type mice; yet, ccr2-null mice did not develop movement-provoked pain behaviors within 8 weeks in a surgical model of OA induced by medial meniscus (DMM) instability [68]. Another study found that the absence of CCR2 strongly suppressed selective inflammatory response genes in the joint with a lower average chondropathy score and delays pain-related behavior DMM [69]. On the contrary, Raghu et al. reported that mice lacking CCR2 were protected against OA by attenuating macrophage accumulation in the synovial joints [70], thus indicating that the CCL2/CCR2 signaling axis preferentially mediates monocyte trafficking and promotes inflammation and tissue damage in OA. These conflicting results might be due to differences in experimental design, including older mice model (20-week-old vs 10-week-old) and duration of OA development (20 weeks after DMM vs 8–12 weeks). Therefore, the function of CCR2 remains unclear in the development of OA, and CCL2/CCR2 inhibition in the treatment of OA should be regarded with caution.

However, there are still some limitations. For instance, diversity and plasticity are hallmarks of macrophages, and the M1/M2 paradigm is a limited attempt to define its complexity. *In vivo*, macrophages respond to environmental cues by acquiring distinct functional phenotypes. In mice, during the progression of the inflammatory response, the M1-to-M2 switch enables macrophages to perform different activities at different stages [71]. Previous studies have also shown that macrophages can undergo dynamic transitions between different functional states with a mixture of M1 and M2 phenotypes [72,73]. In addition, differences in macrophage biology between mice and humans in terms of phenotype, homology, transcription factors, and functions may confound the interpretation of results. For instance, murine and human macrophages express different cell markers [74]. Macrophages from mice or humans also exhibit differential metabolic responses to LPS [75]. Therefore, study results on mice should be interpreted in relation to the latent differences when implementing potential therapeutic approaches in humans. In addition, inflammatory processes may substantially vary between patients. The role of macrophages in OA pathogenesis differs by disease stage and endotype [76]. A clear understanding of the immunopathological patterns of OA is critical for further research.

## 4 Summary and future directions

OA is the main cause of lower-limb disability in the elderly [86]. Age is the leading risk factor for OA. Due to the aging population worldwide, an increasing number of patients are at risk of developing OA, which imposes a tremendous economic burden, including productivity and health care. Macrophages have been identified as the main pathological features of OA. They regulate the immune-inflammatory response of synovial tissues, secrete various inflammatory factors such as TNF-α and IL-1β, promote the infiltration of other inflammatory cells, and directly produce cytokines such as MMPs, which in turn accelerate articular cartilage damage and mediate osteophyte formation upon TGFβ stimulation. The damaged articular cartilage fragments subsequently trigger more macrophage activation, forming a vicious circle. Several studies have highlighted the impact of the phenotypic changes of macrophages in the development of OA [76]. The role of macrophages in synovitis and OA has gradually become the focus of therapeutic interventions. Overall, inhibiting the M1 polarization of macrophages and blocking the expression of TNF-α and MMPs may provide novel insights to guide the clinical treatment of OA.

## References

[1] Glyn-Jones S, Palmer AJR, Agricola R, Price AJ, Vincent TL, Weinans H, et al. Osteoarthritis. Lancet. 2015;386(9991):376–87. 10.1016/S0140-6736(14)60802-3 25748615

[2] Thomas E, Peat G, Croft P. Defining and mapping the person with osteoarthritis for population studies and public health. Rheumatol (United Kingdom). 2014;53(2):338–45. 10.1093/rheumatology/ket346 PMC3894672 24173433

[3] Musumeci G, Aiello FC, Szychlinska MA, Di Rosa M, Castrogiovanni P, Mobasheri A. Osteoarthritis in the XXIst century: Risk factors and behaviours that influence disease onset and progression. Int J Mol Sci. 2015;16(3):6093–112. 10.3390/ijms16036093 PMC4394521 25785564

[4] Sebastian A, Hum NR, McCool JL, Wilson SP, Murugesh DK, Martin KA, et al. Single-cell RNA-Seq reveals changes in immune landscape in post-traumatic osteoarthritis. Front Immunol. 2022;13:938075. 10.3389/fimmu.2022.938075. PMC9373730 35967299

[5] Tay TL, Béchade C, D’Andrea I, St-Pierre M-K, Henry MS, Roumier A, et al. Microglia Gone Rogue: Impacts on Psychiatric Disorders across the Lifespan. Front Mol Neurosci. 2018;10:421. 10.3389/fnmol.2017.00421. PMC5758507 29354029

[6] Robinson WH, Lepus CM, Wang Q, Raghu H, Mao R, Lindstrom TM, et al. Low-grade inflammation as a key mediator of the pathogenesis of osteoarthritis. Nat Rev Rheumatol. 2016;12(10):580–92. 10.1038/nrrheum.2016.136 PMC5500215 27539668

[7] Xie J, Huang Z, Yu X, Zhou L, Pei F. Clinical implications of macrophage dysfunction in the development of osteoarthritis of the knee. Cytokine Growth Factor Rev. 2019;46:36–44. 10.1016/j.cytogfr.2019.03.004 30910350

[8] Khella CM, Horvath JM, Asgarian R, Rolauffs B, Hart ML. Anti-inflammatory therapeutic approaches to prevent or delay post-traumatic osteoarthritis (PTOA) of the knee joint with a focus on sustained delivery approaches. Int J Mol Sci. 2021;22:8005. 10.3390/ijms22158005 PMC8347094 34360771

[9] Calabrese G, Zappal A, Dolcimascolo A, Acquaviva R, Parenti R, Malfa GA. (Scrophulariaceae) Leaf extract evaluated in two in vitro models of inflammation and osteoarthritis. Molecules. 2021;26(17):5392. 10.3390/molecules26175392 PMC8434610 34500824

[10] Conaghan PG, Cook AD, Hamilton JA, Tak PP. Therapeutic options for targeting inflammatory osteoarthritis pain. Nat Rev Rheumatol. 2019;15(6):355–63. 10.1038/s41584-019-0221-y 31068673

[11] Fernandes TL, Gomoll AH, Lattermann C, Hernandez AJ, Bueno DF, Amano MT. Macrophage: A potential target on cartilage regeneration. Front Immunol. 2020;11:111. 10.3389/fimmu.2020.00111. PMC7026000 32117263

[12] Menarim BC, Gillis KH, Oliver A, Ngo Y, Werre SR, Barrett SH, et al. Macrophage activation in the synovium of healthy and osteoarthritic equine. Joints. 2020;7(November):1–14. 10.3389/fvets.2020.568756 PMC7726135 33324696

[13] Kurowska-Stolarska M, Alivernini S. Synovial tissue macrophages: Friend or foe? RMD Open. 2017;3(2):1–10. 10.1136/rmdopen-2017-000527 PMC5729306 29299338

[14] Rayahin JE, Gemeinhart RA. Activation of macrophages in response to biomaterials. Results Probl Cell Differ. Springer; 2017;62:317–51. 10.1007/978-3-319-54090-0_13. 28455715

[15] Gómez-Aristizábal A, Gandhi R, Mahomed NN, Marshall KW, Viswanathan S. Synovial fluid monocyte/macrophage subsets and their correlation to patient-reported outcomes in osteoarthritic patients: A cohort study. Arthritis Res Ther. 2019;21(1):1–10. 10.1186/s13075-018-1798-2 PMC6339358 30658702

[16] Bondeson J, Wainwright SD, Lauder S, Amos N, Hughes CE. The role of synovial macrophages and macrophage-produced cytokines in driving aggrecanases, matrix metalloproteinases, and other destructive and inflammatory responses in osteoarthritis. Arthritis Res Ther. 2006;8(6):1–12. 10.1186/ar2099 PMC1794533 17177994

[17] Jaguin M, Houlbert N, Fardel O, Lecureur V. Polarization profiles of human M-CSF-generated macrophages and comparison of M1-markers in classically activated macrophages from GM-CSF and M-CSF origin. Cell Immunol. 2013 Jan;281(1):51–61. 10.1016/j.cellimm.2013.01.010. 23454681

[18] Murphy EA, Davis JM, McClellan JL, Steiner JL, Jung D, Carmichael MD, et al. Linking tumor associated macrophages, inflammation, and intestinal tumorigenesis: Role of MCP-1. FASEB J. 2012 Apr 1;26(S1):479.5. 10.1096/fasebj.26.1_supplement.479.5. PMC3517651 23019193

[19] Atukorala I, Kwoh CK, Guermazi A, Roemer FW, Boudreau RM, Hannon MJ, et al. Synovitis in knee osteoarthritis: A precursor of disease? Ann Rheum Dis. 2016;75(2):390–5. 10.1136/annrheumdis-2014-205894 PMC4916836 25488799

[20] Bondeson J, Blom AB, Wainwright S, Hughes C, Caterson B, Van Den Berg WB. The role of synovial macrophages and macrophage-produced mediators in driving inflammatory and destructive responses in osteoarthritis. Arthritis Rheum. 2010;62(3):647–57. 10.1002/art.27290 20187160

[21] Mathiessen A, Conaghan PG. Synovitis in osteoarthritis: Current understanding with therapeutic implications. Arthritis Res Ther. 2017;19(1):1–9. 10.1186/s13075-017-1229-9. PMC5289060 28148295

[22] Wang CQ, Udupa KB, Xiao H, Lipschitz DA. Effect of age on marrow macrophage number and function. Aging Clin Exp Res. 1995;7(5):379–84. 10.1007/BF03324349 8719605

[23] Jackaman C, Radley-Crabb HG, Soffe Z, Shavlakadze T, Grounds MD, Nelson DJ. Targeting macrophages rescues age-related immune deficiencies in C57BL/6J geriatric mice. Aging Cell. 2013;12(3):345–57. 10.1111/acel.12062 23442123

[24] Hearps AC, Martin GE, Angelovich TA, Cheng WJ, Maisa A, Landay AL, et al. Aging is associated with chronic innate immune activation and dysregulation of monocyte phenotype and function. Aging Cell. 2012;11(5):867–75. 10.1111/j.1474-9726.2012.00851.x 22708967

[25] Chelvarajan RL, Collins SM, Van Willigen JM, Bondada S. The unresponsiveness of aged mice to polysaccharide antigens is a result of a defect in macrophage function. J Leukoc Biol. 2005;77(4):503–12. http://doi.wiley.com/10.1189/jlb.0804449. 10.1189/jlb.0804449 15629885

[26] Mancuso P, McNish RW, Peters-Golden M, Brock TG. Evaluation of phagocytosis and arachidonate metabolism by alveolar macrophages and recruited neutrophils from F344xBN rats of different ages. Mech Ageing Dev. 2001;122(15):1899–913. 10.1016/s0047-6374(01)00322-0 11557288

[27] Aprahamian T, Takemura Y, Goukassian D, Walsh K. Ageing is associated with diminished apoptotic cell clearance in vivo. Clin Exp Immunol. 2008;152(3):448–55. 10.1111/j.1365-2249.2008.03658.x PMC2453212 18422728

[28] Griffin TM, Scanzello CR. Innate inflammation and synovial macrophages in osteoarthritis pathophysiology. Clinical Exp Rheumatol. 2019;37:57–63. PMC6842324 31621560

[29] Zhang H, Cai D, Bai X. Macrophages regulate the progression of osteoarthritis. Osteoarthr Cartil. 2020;28(5):555–61. 10.1016/j.joca.2020.01.007. 31982565

[30] Utomo L, Bastiaansen-Jenniskens YM, Verhaar JAN, van Osch GJVM. Cartilage inflammation and degeneration is enhanced by pro-inflammatory (M1) macrophages in vitro, but not inhibited directly by anti-inflammatory (M2) macrophages. Osteoarthr Cartil. 2016;24(12):2162–70. 10.1016/j.joca.2016.07.018 27502245

[31] Edwards JP, Zhang X, Frauwirth KA, Mosser DM. Biochemical and functional characterization of three activated macrophage populations. J Leukoc Biol. 2006 Dec 1;80(6):1298–307. 10.1189/jlb.0406249. PMC2642590 16905575

[32] Liu B, Zhang M, Zhao J, Zheng M, Yang H. Imbalance of M1/M2 macrophages is linked to severity level of knee osteoarthritis. Exp Ther Med. 2018;16(6):5009–14. 10.3892/etm.2018.6852 PMC6256852 30546406

[33] Roszer T. Understanding the mysterious M2 macrophage through activation markers and effector mechanisms. Mediators Inflamm. 2015;2015:16–8. 10.1155/2015/816460 PMC4452191 26089604

[34] Gordon S, Taylor PR. Monocyte and macrophage heterogeneity. Nat Rev Immunol. 2005;5(12):953–64. 10.1038/nri1733 16322748

[35] Orekhov AN, Orekhova VA, Nikiforov NG, Myasoedova VA, Grechko AV, Romanenko EB, et al. Monocyte differentiation and macrophage polarization. Vessel Plus. 2019;3:10. 10.20517/2574-1209.2019.04.

[36] Tanaka Y, Otsuka T, Hotokebuchi T, Miyahara H, Nakashima H, Kuga S, et al. Effect of IL-10 on collagen-induced arthritis in mice. Inflamm Res. 1996;45(6):283–8. 10.1007/BF02280992. 8814459

[37] Mosmann VR, Cherwinski H, Bond MW, Giedlin MA, Coffman RL. Two types of murine helper T cell clone. I. Definition according to profiles of lymphokine activities and secreted proteins. J Immunol. 2016;136(7):2348–57. http://www.jimmunol.org/content/136/7/2348%5Cnhttp://www.jimmunol.org/content/136/7/2348. 2419430

[38] Zhang H, Lin C, Zeng C, Wang Z, Wang H, Lu J, et al. Synovial macrophage M1 polarisation exacerbates experimental osteoarthritis partially through R-spondin-2. Ann Rheum Dis. 2018 Jul 10;77(10):1524–34. 10.1136/annrheumdis-2018-213450 29991473

[39] Blom AB, van Lent PLEM, Holthuysen AEM, van der Kraan PM, Roth J, van Rooijen N, et al. Synovial lining macrophages mediate osteophyte formation during experimental osteoarthritis. Osteoarthr Cartil. 2004;12(8):627–35. 10.1016/j.joca.2004.03.003 15262242

[40] Thomson A, Hilkens CMU. Synovial macrophages in osteoarthritis: The key to understanding pathogenesis? Front Immunol. 2021;12(June):1–9. 10.3389/fimmu.2021.678757 PMC8239355 34211470

[41] Sampaio C, Sindrup SH, Stauffer JW, Steigerwald I, Stewart J, Tobias J, et al. Direct in vivo evidence of activated macrophages in human osteoarthritis. 2015;155(9):1683–95.

[42] Roemer FW, Kassim Javaid M, Guermazi A, Thomas M, Kiran A, Keen R, et al. Anatomical distribution of synovitis in knee osteoarthritis and its association with joint effusion assessed on non-enhanced and contrast-enhanced MRI. Osteoarthr Cartil. 2010;18(10):1269–74. 10.1016/j.joca.2010.07.008. 20691796

[43] Chen Y, Jiang W, Yong H, He M, Yang Y, Deng Z, et al. Macrophages in osteoarthritis: Pathophysiology and therapeutics. Am J Transl Res. 2020;12(1):261–8. PMC7013211 32051751

[44] Topoluk N, Steckbeck K, Siatkowski S, Burnikel B, Tokish J, Mercuri J. Amniotic mesenchymal stem cells mitigate osteoarthritis progression in a synovial macrophage-mediated in vitro explant coculture model. J Tissue Eng Regen Med. 2018;12(4):1097–110. 10.1002/term.2610 PMC5906145 29131526

[45] Li G, Yin J, Gao J, Cheng TS, Pavlos NJ, Zhang C, et al. Subchondral bone in osteoarthritis: Insight into risk factors and microstructural changes. Arthritis Res Ther. 2013;15(6):223. http://arthritis-research.com/content/15/6/223%5Cnhttp://ovidsp.ovid.com/ovidweb.cgi?T=JS&PAGE=reference&D=emed11&NEWS=N&AN=2013776791. 10.1186/ar4405 PMC4061721 24321104

[46] Utomo L, van Osch GJVM, Bayon Y, Verhaar JAN, Bastiaansen-Jenniskens YM. Guiding synovial inflammation by macrophage phenotype modulation: An in vitro study towards a therapy for osteoarthritis. Osteoarthr Cartil. 2016;24(9):1629–38. 10.1016/j.joca.2016.04.013. 27095417

[47] Sabokbar A, Crawford R, Murray DW, Athanasou NA. Macrophage-osteoclast differentiation and bone resorption in osteoarthrotic subchondral acetabular cysts. Acta Orthop Scand. 2000;71(3):255–61. 10.1080/000164700317411843 10919296

[48] Adamopoulos IE, Wordsworth PB, Edwards JR, Ferguson DJ, Athanasou NA. Osteoclast differentiation and bone resorption in multicentric reticulohistiocytosis. Hum Pathol. 2006;37(9):1176–85. 10.1016/j.humpath.2006.04.007 16938523

[49] Lam J, Takeshita S, Barker JE, Kanagawa O, RossFP SLT. TNF-alpha induces osteoclastogenesis by direct stimulation of macrophages exposed to permissive levels of RANK ligand. J Clin Invest. 2000;106(12):7. 10.1172/JCI11176 PMC387259 11120755

[50] van Meurs J, van Lent P, Holthuysen A, Lambrou D, Bayne E, Singer I, et al. Active matrix metalloproteinases are present in cartilage during immune complex-mediated arthritis: A pivotal role for stromelysin-1 in cartilage destruction. J Immunol. 1999;163(10):5633–9. http://www.ncbi.nlm.nih.gov/pubmed/10553093. 10553093

[51] Blom AB, Van Lent PL, Libregts S, Holthuysen AE, Van Der Kraan PM, Van Rooijen N, et al. Crucial role of macrophages in matrix metalloproteinase-mediated cartilage destruction during experimental osteoarthritis: Involvement of matrix metalloproteinase 3. Arthritis Rheum. 2007;56(1):147–57. 10.1002/art.22337 17195217

[52] Blom AB, van Lent PLEM, Holthuysen AEM, van der Kraan PM, Roth J, van Rooijen N, et al. Synovial lining macrophages mediate osteophyte formation during experimental osteoarthritis. Osteoarthr Cartil. 2004;12(8):627–35. 10.1016/j.joca.2004.03.003 15262242

[53] Wu CL, McNeill J, Goon K, Little D, Kimmerling K, Huebner J, et al. Conditional macrophage depletion increases inflammation and does not inhibit the development of osteoarthritis in obese macrophage fas-induced apoptosis–transgenic mice. Arthritis Rheumatol. 2017;69(9):1772–83. 10.1002/art.40161 PMC5611814 28544542

[54] Bailey KN, Furman BD, Zeitlin J, Kimmerling KA, Wu CL, Guilak F, et al. Intra-articular depletion of macrophages increases acute synovitis and alters macrophage polarity in the injured mouse knee. Osteoarthr Cartil. 2020;28(5):626–38. 10.1016/j.joca.2020.01.015 PMC8963860 32044353

[55] Chamberlain CS, Leiferman EM, Frisch KE, Wang S, Yang X, van Rooijen N, et al. The influence of macrophage depletion on ligament healing. Connect Tissue Res. 2011 Jun 30;52(3):203–11. https://onlinelibrary.wiley.com/doi/10.1111/j.1524-475X.2011.00682.x. 10.3109/03008207.2010.511355 PMC3110150 21117894

[56] Lv Z, Xu X, Sun Z, Yang YX, Guo H, Li J, et al. TRPV1 alleviates osteoarthritis by inhibiting M1 macrophage polarization via Ca2+/CaMKII/Nrf2 signaling pathway. Cell Death Dis. 2021;12(6):504. 10.1038/s41419-021-03792-8. PMC8131608 34006826

[57] Tian Z, Zeng F, Zhao C, Dong S. Angelicin alleviates post-trauma osteoarthritis progression by regulating macrophage polarization via STAT3 signaling pathway. Front Pharmacol. 2021;12(June):1–11. 10.3389/fphar.2021.669213 PMC8223070 34177582

[58] Dai M, Sui B, Xue Y, Liu X, Sun J. Cartilage repair in degenerative osteoarthritis mediated by squid type II collagen via immunomodulating activation of M2 macrophages, inhibiting apoptosis and hypertrophy of chondrocytes. Biomaterials. 2018;180:91–103. 10.1016/j.biomaterials.2018.07.011. 30031224

[59] Zhang J, Rong Y, Luo C, Cui W. Bone marrow mesenchymal stem cell-derived exosomes prevent osteoarthritis by regulating synovial macrophage polarization. Aging. 2020;12(24):25138–52. 10.18632/aging.104110 PMC7803581 33350983

[60] Young L, Katrib A, Cuello C, Vollmer-Conna U, Bertouch JV, Roberts-Thomson PJ, et al. Effects of intraarticular glucocorticoids on macrophage infiltration and mediators of joint damage in osteoarthritis synovial membranes: Findings in a double-blind, placebo-controlled study. Arthritis Rheum. 2001;44(2):343–50. 10.1002/1529-0131(200102)44:2<343::AID-ANR52>3.0.CO;2-Q 11229465

[61] Pasquali Ronchetti I, Guerra D, Taparelli F, Boraldi F, Bergamini G, Mori G, et al. Morphological analysis of knee synovial membrane biopsies from a randomized controlled clinical study comparing the effects of sodium hyaluronate (Hyalgan®) and methylprednisolone acetate (Depomedrol®) in osteoarthritis. Rheumatology. 2001 Feb;40(2):158–69. https://academic.oup.com/rheumatology/article-lookup/doi/10.1093/rheumatology/40.2.158. 10.1093/rheumatology/40.2.158 11257152

[62] Daghestani HN, Pieper CF, Kraus VB. Soluble macrophage biomarkers indicate inflammatory phenotypes in patients with knee osteoarthritis. Arthritis Rheumatol. 2015;67(4):956–65. 10.1002/art.39006 PMC4441094 25544994

[63] Zhou F, Mei J, Han X, Li H, Yang S, Wang M, et al. Kinsenoside attenuates osteoarthritis by repolarizing macrophages through inactivating NF-κB/MAPK signaling and protecting chondrocytes. Acta Pharm Sin B. 2019;9(5):973–85. 10.1016/j.apsb.2019.01.015 PMC6804452 31649847

[64] Lee H, Kim H, Lee Y, Park K, Lee B, Kim S, et al. INVOSSA-K induces an anti-inflammatory intra-articular environment in a rat MIA model via macrophage polarization. Osteoarthr Cartil. 2019;27(2019):S376–7. 10.1016/j.joca.2019.02.371.

[65] Cosenza S, Ruiz M, Toupet K, Jorgensen C, Noël D. Mesenchymal stem cells derived exosomes and microparticles protect cartilage and bone from degradation in osteoarthritis. Sci Rep. 2017;7(1):1–12. 10.1038/s41598-017-15376-8 PMC5701135 29176667

[66] Kong L, Zheng LZ, Qin L, Ho KKW. Role of mesenchymal stem cells in osteoarthritis treatment. J Orthop Transl. 2017;9:89–103. 10.1016/j.jot.2017.03.006. PMC5822967 29662803

[67] Chahal J, Gómez-Aristizábal A, Shestopaloff K, Bhatt S, Chaboureau A, Fazio A, et al. Bone Marrow Mesenchymal Stromal Cell Treatment in Patients with Osteoarthritis Results in Overall Improvement in Pain and Symptoms and Reduces Synovial Inflammation. Stem Cells Transl Med. 2019;8(8):746–57. 10.1002/sctm.18-0183 PMC6646697 30964245

[68] Miller RE, Tran PB, Das R, Ghoreishi-Haack N, Ren D, Miller RJ, et al. CCR2 chemokine receptor signaling mediates pain in experimental osteoarthritis. Proc Natl Acad Sci U S A. 2012;109(50):20602–7. 10.1073/pnas.1209294110 PMC3528555 23185004

[69] Miotla Zarebska J, Chanalaris A, Driscoll C, Burleigh A, Miller RE, Malfait AM, et al. CCL2 and CCR2 regulate pain-related behaviour and early gene expression in post-traumatic murine osteoarthritis but contribute little to chondropathy. Osteoarthr Cartil. 2017;25(3):406–12. 10.1016/j.joca.2016.10.008. PMC5358501 27746376

[70] Raghu H, Lepus CM, Wang Q, Wong HH, Lingampalli N, Oliviero F, et al. CCL2/CCR2, but not CCL5/CCR5, mediates monocyte recruitment, inflammation and cartilage destruction in osteoarthritis. Ann Rheum Dis. 2017;76(5):914–22. 10.1136/annrheumdis-2016-210426 PMC5834918 27965260

[71] Vogel DYS, Vereyken EJF, Glim JE, Heijnen PDAM, Moeton M, van der Valk P, et al. Macrophages in inflammatory multiple sclerosis lesions have an intermediate activation status. J Neuroinflammation. 2013;10(1):809. 10.1186/1742-2094-10-35. PMC3610294 23452918

[72] Mills CD, Kincaid K, Alt JM, Heilman MJ, Hill AM. M-1/M-2 Macrophages and the Th1/Th2 Paradigm. J Immunol. 2017 Oct;199(7):2194–201. 10.4049/jimmunol.1701141. 28923981

[73] Mills CD, Ley K. M1 and M2 macrophages: The chicken and the egg of immunity. J Innate Immun. 2014;6(6):716–26. https://europepmc.org/articles/PMC4429858. 10.1159/000364945 PMC4429858 25138714

[74] Nayak DK, Mendez O, Bowen S, Mohanakumar T. Isolation and in vitro culture of murine and human alveolar macrophages. J Vis Exp. 2018;134:57287. 10.3791/57287. PMC6100701 29733312

[75] Vijayan V, Pradhan P, Braud L, Fuchs HR, Gueler F, Motterlini R, et al. Human and murine macrophages exhibit differential metabolic responses to lipopolysaccharide – A divergent role for glycolysis. Redox Biol. 2019;22(January):101147. 10.1016/j.redox.2019.101147. PMC6396203 30825774

[76] Mushenkova NV, Nikiforov NG, Shakhpazyan NK, Orekhova VA, Sadykhov NK, Orekhov AN. Phenotype diversity of macrophages in osteoarthritis: implications for development of macrophage modulating therapies. Int J Mol Sci. 2022;23(15):8381. 10.3390/ijms23158381 PMC9369350 35955514

[77] Lee J, Lee J, Lee S, Yoo SA, Kim KM, Kim WU, et al. Genetic deficiency of nuclear factor of activated T cells 5 attenuates the development of osteoarthritis in mice. Jt Bone Spine. 2022;89(1):105273. 10.1016/j.jbspin.2021.105273. 34537377

[78] Xie JW, Wang Y, Xiao K, Xu H, Luo ZY, Li L, et al. Alpha defensin-1 attenuates surgically induced osteoarthritis in association with promoting M1 to M2 macrophage polarization. Osteoarthr Cartil. 2021;29(7):1048–59. 10.1016/j.joca.2021.04.006 33892137

[79] Ma Y, Yang H, Zong X, Wu J, Ji X, Liu W, et al. Artificial M2 macrophages for disease-modifying osteoarthritis therapeutics. Biomaterials. 2021;274(November 2020):120865. 10.1016/j.biomaterials.2021.120865. 33991950

[80] Mahon OR, Kelly DJ, McCarthy GM, Dunne A. Osteoarthritis-associated basic calcium phosphate crystals alter immune cell metabolism and promote M1 macrophage polarization. Osteoarthr Cartil. 2020;28(5):603–12. 10.1016/j.joca.2019.10.010. 31730805

[81] Barreto G, Senturk B, Colombo L, Brück O, Neidenbach P, Salzmann G, et al. Lumican is upregulated in osteoarthritis and contributes to TLR4-induced pro-inflammatory activation of cartilage degradation and macrophage polarization. Osteoarthr Cartil. 2020;28(1):92–101. 10.1016/j.joca.2019.10.011. 31715293

[82] Lee KMC, Prasad V, Achuthan A, Fleetwood AJ, Hamilton JA, Cook AD. Targeting GM-CSF for collagenase-induced osteoarthritis pain and disease in mice. Osteoarthr Cartil. 2020;28(4):486–91. 10.1016/j.joca.2020.01.012. 32028021

[83] Lin X, Wang W, McDavid A, Xu H, Boyce BF, Xing L. The E3 ubiquitin ligase Itch limits the progression of post-traumatic osteoarthritis in mice by inhibiting macrophage polarization. Osteoarthr Cartil. 2021;29(8):1225–36. 10.1016/j.joca.2021.04.009 PMC8319075 33940137

[84] Flannery CR, Seaman SA, Buddin KE, Nasert MA, Semler EJ, Kelley KL, et al. A novel placental tissue biologic, PTP-001, inhibits inflammatory and catabolic responses in vitro and prevents pain and cartilage degeneration in a rat model of osteoarthritis. Osteoarthr Cartil. 2021;29(8):1203–12. 10.1016/j.joca.2021.03.022. 34023528

[85] von Kaeppler EP, Wang Q, Raghu H, Bloom MS, Wong H, Robinson WH. Interleukin 4 promotes anti-inflammatory macrophages that clear cartilage debris and inhibits osteoclast development to protect against osteoarthritis. Clin Immunol. 2021;229(June):108784. 10.1016/j.clim.2021.108784. 34126239

[86] Samavedi S, Diaz-Rodriguez P, Erndt-Marino JD, Hahn MS. A three-dimensional chondrocyte-macrophage coculture system to probe inflammation in experimental osteoarthritis. Tissue Eng Part A. 2017;23(3–4):101–14. http://online.liebertpub.com/doi/10.1089/ten.tea.2016.0007. 10.1089/ten.tea.2016.0007 PMC5312455 27736317

